# Elevated Serum KIM-1 in Sepsis Correlates with Kidney Dysfunction and the Severity of Multi-Organ Critical Illness

**DOI:** 10.3390/ijms25115819

**Published:** 2024-05-27

**Authors:** Jonathan Frederik Brozat, Neval Harbalioğlu, Philipp Hohlstein, Samira Abu Jhaisha, Maike Rebecca Pollmanns, Jule Katharina Adams, Theresa Hildegard Wirtz, Karim Hamesch, Eray Yagmur, Ralf Weiskirchen, Frank Tacke, Christian Trautwein, Alexander Koch

**Affiliations:** 1 Department for Gastroenterology, Metabolic Disorders and Intensive Care Medicine, University Hospital RWTH Aachen, RWTH Aachen, Pauwelsstraße 30, 52074 Aachen, Germany; jonathan-frederik.brozat@charite.de (J.F.B.); nharbalioglu@ukaachen.de (N.H.); phohlstein@ukaachen.de (P.H.); sabujhaisha@ukaachen.de (S.A.J.); mpollmanns@ukaachen.de (M.R.P.); jadams@ukaachen.de (J.K.A.); thwirtz@ukaachen.de (T.H.W.); khamesch@ukaachen.de (K.H.); trautwein.christian@gmx.net (C.T.); 2 Department of Hepatology and Gastroenterology, Charité—Universitätsmedizin Berlin, Campus Virchow-Klinikum (CVK) and Campus Charité Mitte (CCM), Augustenburger Platz 1, 13353 Berlin, Germany; frank-tacke@charite.de; 3 Institute of Laboratory Medicine, Western Palatine Hospital, Hellmut-Hartert-Straße 1, 67655 Kaiserslautern, Germany; eyagmur@ukaachen.de; 4 Institute of Molecular Pathobiochemistry, Experimental Gene Therapy and Clinical Chemistry, University Hospital Aachen, RWTH Aachen, Pauwelsstraße 30, 52074 Aachen, Germany; rweiskirchen@ukaachen.de

**Keywords:** KIM-1, TIM-1, intensive care unit, kidney dysfunction, organ failure, sepsis, biomarker

## Abstract

The kidney injury molecule (KIM)-1 is shed from proximal tubular cells in acute kidney injury (AKI), relaying tubular epithelial proliferation. Additionally, KIM-1 portends complex immunoregulation and is elevated after exposure to lipopolysaccharides. It thus may represent a biomarker in critical illness, sepsis, and sepsis-associated AKI (SA-AKI). To characterise and compare KIM-1 in these settings, we analysed KIM-1 serum concentrations in 192 critically ill patients admitted to the intensive care unit. Irrespective of kidney dysfunction, KIM-1 serum levels were significantly higher in patients with sepsis compared with other critical illnesses (191.6 vs. 132.2 pg/mL, *p* = 0.019) and were highest in patients with urogenital sepsis, followed by liver failure. Furthermore, KIM-1 levels were significantly elevated in critically ill patients who developed AKI within 48 h (273.3 vs. 125.8 pg/mL, *p* = 0.026) or later received renal replacement therapy (RRT) (299.7 vs. 146.3 pg/mL, *p* < 0.001). KIM-1 correlated with markers of renal function, inflammatory parameters, hematopoietic function, and cholangiocellular injury. Among subcomponents of the SOFA score, KIM-1 was elevated in patients with hyperbilirubinaemia (>2 mg/dL, *p* < 0.001) and thrombocytopenia (<150/nL, *p* = 0.018). In univariate and multivariate regression analyses, KIM-1 predicted sepsis, the need for RRT, and multi-organ dysfunction (MOD, SOFA > 12 and APACHE II ≥ 20) on the day of admission, adjusting for relevant comorbidities, bilirubin, and platelet count. Additionally, KIM-1 in multivariate regression was able to predict sepsis in patients without prior (CKD) or present (AKI) kidney injury. Our study suggests that next to its established role as a biomarker in kidney dysfunction, KIM-1 is associated with sepsis, biliary injury, and critical illness severity. It thus may offer aid for risk stratification in these patients.

## 1. Introduction

Sepsis and septic shock continue to be the leading causes of death among critically ill patients [1,2]. Culminating in severe organ dysfunction, sepsis is predicated on a dysregulated immune response to infection. Early identification, diagnosis, and treatment of sepsis are the most potent clinical crossroads in determining later survival but are also notoriously difficult [3,4]. Thus, easy risk stratification through clinical presentation, common scoring systems (sepsis-related organ failure assessment, SOFA) and biological markers are ever useful in objectifying symptoms into a timely diagnosis [3].

Renal clearance is closely linked to microcirculatory dysfunction and dysregulated inflammatory responses. As a result, kidney failure occurs early in the clinical course of sepsis, portraying worsened prognosis and increased mortality [5,6,7]. However, predicting incipient (subclinical) sepsis-associated acute kidney injury (SA-AKI) prior to actual dysfunction has been difficult. Only a few markers, e.g., soluble urokinase receptor (suPAR), may be suitable to predict AKI and/or sepsis [8,9,10,11].

The kidney injury molecule 1 (KIM-1), also known as T-cell immunoglobulin mucin receptor 1 (TIM-1), is a transmembrane protein mainly found on epithelial cells of the proximal renal tubule and is hardly detected in healthy kidneys [12,13,14]. The extracellular domain contains a unique hydrophobic metal-ion-dependent binding site called MILIBS [15]. This MILIBS is uniquely able to identify phosphatidylserine, e.g., on apoptotic leaflets, thus acting as a non-myeloid scavenger receptor [14,16,17,18].

Even though the amount of KIM-1 transcript in the kidney is 10 times higher than in other organs, immunological studies have demonstrated significant levels of KIM-1 protein expression in the intestine and biliary system [19,20,21,22]. Of note, conflicting data exist regarding the presence of KIM-1 on hepatocytes [21,23,24,25,26].

Depending on differential splicing, KIM-1 can be expressed on cell surfaces, cluster in the cytoplasm, or be shed into extracellular compartments such as urine and blood [27].

It thus portrays a wide variety of functions. Available data suggest that KIM-1 influences multiple regulatory axes within lymphatic cell lineages [28]. KIM-1 on CD4+ T cells can directly regulate Th2-dependent immunomodulation by releasing interleukin 10 [29,30]. In addition, KIM-1 acts as a pattern recognition receptor on invariant natural killer cells (iNKT), mediating iNKT activation. It has been described as both a co-receptor to the T-cell receptor complex (TCR) [31] and a co-factor in anti-inflammatory IL10-release and Th1 suppression [32,33]. Therefore, it may play a role in relaying anti-inflammatory influences in dysregulated immune responses.

As mentioned, KIM-1 is rarely expressed in unchallenged kidney tissue but is amplified and shed into the renal tube in acute injury. In response to various causes of AKI, KIM-1 has been shown to possess anti-inflammatory effects. It further transforms non-phagocytes into protective semi-professional phagocytic cells, allowing rapid clearance of apoptotic cells [25]. Increased KIM-1 expression has been shown to reduce acute kidney injury in different animal models [14]. Injury-dependent blood and urine KIM-1 have been extensively studied as markers for allograft rejection and other acute injuries to the proximal tubule [34,35]. In chronic kidney dysfunction (CKD), KIM-1 has been found to be elevated in a plethora of aetiologies [36,37,38].

Lastly, serum KIM-1 has been shown to be elevated in response to lipopolysaccharides (LPS)-induced inflammation in humans [39]. It remains uncertain whether this elevation is an immunoregulative response or a sign of subclinical kidney injury.

Given its role in kidney dysfunction, deep integration in immunoregulation, and response to inflammatory stimuli, KIM-1 may be a promising biomarker in sepsis and critical illness. In contrast to urine homologs, serum KIM-1 in sepsis and SA-AKI has been less well investigated.

This study therefore aimed to investigate the functional correlation between critical illness, sepsis, SA-AKI, and serum KIM-1.

## 2. Results

### 2.1. Sepsis and Septic Shock Are Associated with a Higher Need for Renal Replacement Therapy and Invasive Ventilation Compared with Other Critical Illnesses

Out of 192 patients consecutively admitted to the intensive care unit at University Hospital RWTH Aachen, 125 patients (65%) had sepsis or septic shock, as defined according to the Sepsis-3 consensus criteria (Table 1) [1]. The most common infection sites were pulmonary (55.2%, n = 69), followed by abdominal (15.2%, n = 19), urogenital (8%, n = 10), or other (21.6%, n = 27) (Table 2).

A control group was also established, consisting of patients admitted to the ICU for critical illness unrelated to sepsis (n = 67). The main reasons for admission to critical care in this group were cardiovascular disease (19.4%, n = 13), advanced liver disease (19.4%, n = 13), exacerbated COPD (14.9%, n = 10), and other reasons (46.3%, n = 31) (Table 2). The age, body weight, and sex distribution were similar between the two groups. The presence of comorbid conditions, such as chronic obstructive pulmonary disease (COPD), chronic heart failure, cardiovascular diseases (CVD), diabetes (DM), chronic kidney disease, and hypertension (AHT), was evenly distributed. However, patients admitted due to sepsis had slightly lower rates of alcohol use disorder and previously known chronic liver disease (8% vs. 19%, *p* = 0.018 and 7% vs. 16%, *p* = 0.047, Table 1). The median Charlson comorbidity index (CCI) reflected the comparability, with rates of four points for either cohort (for all other parameters, see Table 1).

Expectedly, patients admitted due to sepsis had significantly higher sepsis-related organ failure assessment (SOFA) and acute physiology and chronic health disease classification system (APACHE)-II scores (11 [6.75; 14.25] vs. 7 [3; 11], *p* = 0.012 and 18 [11; 24] vs. 16 [8; 21], *p* = 0.039, respectively) (Table 3) [40]. Additionally, septic patients had a significantly higher need for mechanical ventilation (n = 92 [73.6%] vs. 38 [56.7%], *p* = 0.024) as well as a longer duration of mechanical ventilation (181 [0; 480] vs. 27 [0; 176] days, *p* = 0.002) than non-septic patients. Furthermore, septic patients were more likely to need renal replacement therapy (RRT, 38 [30.3%] vs. 12 [17.9%] patients, *p* = 0.045) and required dialysis/hemofiltration longer than patients without sepsis. The demand for vasopressors (i.e., norepinephrine) on day 0 was elevated in patients with sepsis compared with non-septic patients (59.35 vs. 0 µg/kg/h, *p* = 0.005). Death in the ICU occurred in 30.4% (38) of cases in the sepsis cohort and 20.9% (14) in the non-sepsis cohort (*p* = 0.214). Patients with sepsis showed higher case fatality rates at 30 and 90 days, as well as one year, but rates did not differ significantly from patients with other critical illnesses.

### 2.2. Serum (s)KIM-1 Is Elevated in Patients with Sepsis and Septic Shock at ICU Admission

**Figure 1:**
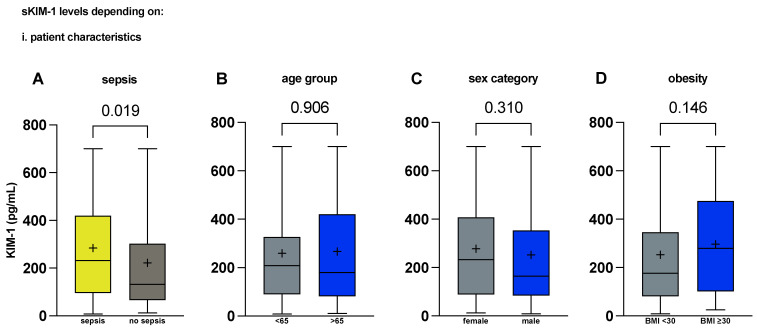
Serum KIM-1 is elevated in sepsis and independent from demographics. Serum KIM-1 concentrations on admission day in critically ill patients (**A**) with sepsis/septic shock compared with patients with other critical illness (n = 125 vs. 67); (**B**) compared between age groups (n = 96 vs. 96); (**C**) compared between sex categories (n = 79 vs. 113); (**D**) dependent on obesity with BMI > 30 (n = 149 vs. 43). *p* values reported are from the Mann–Whitney U test and were considered statistically significant at *p* < 0.05. Medians with first and third quartile are shown. Means are shown as +. Whiskers represent lowest and highest value. BMI, body mass index. KIM-1, kidney injury molecule-1.

Strikingly, alongside established laboratory parameters of infection/inflammation (white blood cell count, C-reactive protein, procalcitonin, interleukin-6), serum (s)KIM-1 concentrations at admission were significantly higher in patients with sepsis than in patients without (191.6 [85.8; 388.9] vs. 132.2 [66.1; 302.3] pg/mL, *p* = 0.019, Table 1 and Figure 1A). Creatinine levels at admission showed a trend towards significance but did not reach it between the two cohorts (Table 1). Other, less confounded, delayed, and sensitive parameters for renal dysfunction, such as cystatin C and urea levels, were already elevated at admission, demonstrating that the patients with sepsis were prone to experiencing renal dysfunction (Table 1).

### 2.3. Elevated sKIM-1 Concentrations Are Associated with CKD

**Figure 2:**
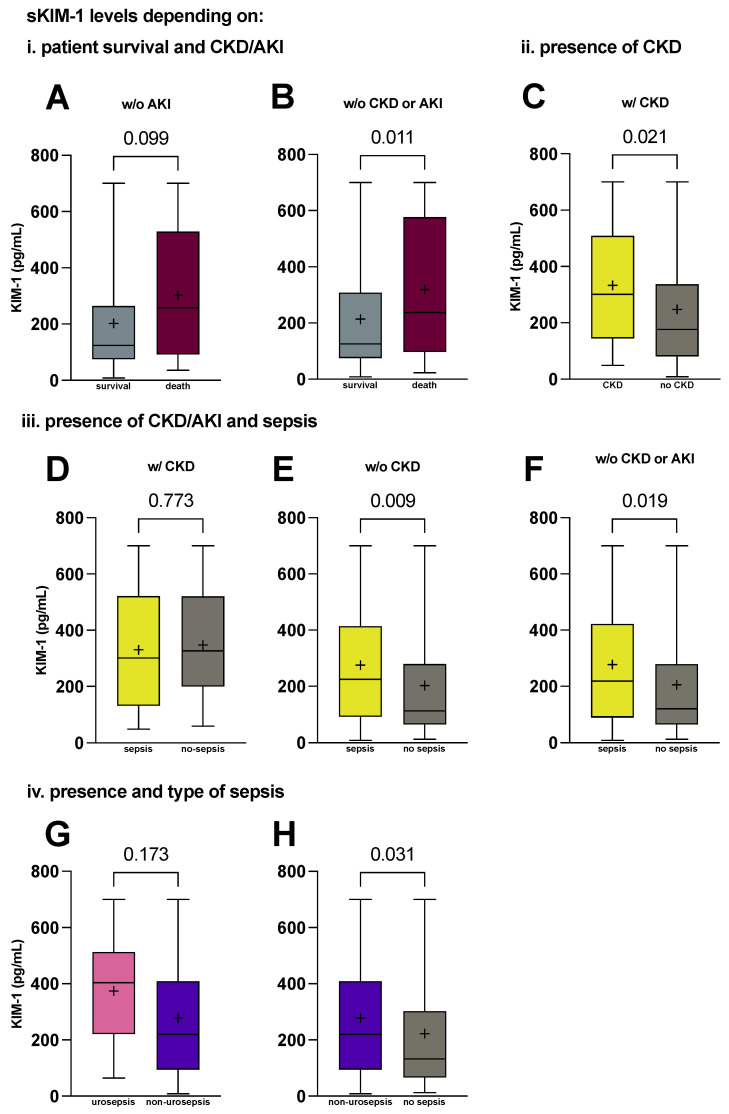
Serum KIM-1 elevation is associated with ICU mortality and independent from CKD or AKI on admission day. Serum KIM-1 concentrations on admission day in critically ill patients (**A**) that had no AKI (KDIGO classification) after 48 h (day 2) and survived the ICU versus patients that died (n = 55 vs. 20); (**B**) that had neither chronic kidney disease nor AKI on day 2 (KDIGO classification) and survived the ICU versus patients that died (n = 92 vs. 39); (**C**) with known chronic kidney disease and without (n = 33 vs. 159); (**D**) with CKD and sepsis compared with patients with other critical illness (n = 23 vs. 9); (**E**) without CKD and sepsis compared with patients with other critical illness (n = 100 vs. 58); (**F**) without CKD or AKI on day 2 (KDIGO classification) and sepsis/septic shock compared with patients with other critical illness (no sepsis) (n = 84 vs. 51); (**G**) compared between urosepsis and other types of sepsis (pulmonal, abdominal, other) (n = 10 vs. 114); (**H**) compared between patients with sepsis other than urogenital aetiology (non-urosepsis) and patients without sepsis (n = 114 vs. 67). *p* values reported are from the Mann–Whitney U test and were considered statistically significant at *p* < 0.05. Medians with first and third quartile are shown. Means are shown as +. Whiskers represent lowest and highest value. AKI, acute kidney injury. CKD, chronic kidney disease. KIM-1, kidney injury molecule-1. w/, with. w/o, without.

To dissect KIM-1 elevation, we performed extensive subgroup analyses. In these cohorts of critically ill patients, there were no differences in KIM-1 levels based on sex or age, and body weight did not have an impact (Figure 1B–D). Of all concomitant diseases present in the cohorts, stratifying by documented CKD yielded a significant difference (301 vs. 176.3 pg/mL, *p* = 0.021, Figure 2C). Interestingly, documented COPD also offered a significant difference (104.41 vs. 218.7, *p* = 0.035, for a complete subgroup overview please also note Supplementary Figure S1). Investigating all comorbidities, median KIM-1 serum concentrations were highest in patients with known chronic kidney disease, followed by heart failure and documented hypertension. Patients with COPD had the lowest serum KIM-1 concentrations (Table 4).

### 2.4. KIM-1 Serum Increase in Sepsis Is Higher in Renal Dysfunction, but Independent from CKD and AKI

Patients with sepsis at admission demonstrated elevated levels of renal retention (cystatin C, urea) and inflammatory parameters. Furthermore, we found that known CKD was associated with a higher KIM-1 serum concentration on admission, regardless of the presence of sepsis. We thus compared subgroups dependent on the presence of acute or (documented) chronic kidney failure.

Interestingly, even though KIM-1 at admission day did not differ between patients who subsequently died in the ICU, KIM-1 showed a trend in patients without AKI after 48 h (257.5 vs. 124 pg/mL, *p* = 0.099, Figure 2A). This trend became significant when we specifically analysed patients without any renal dysfunction (CKD or AKI after 48 h) (237.1 vs. 125.5 pg/mL, *p* = 0.011 Figure 2B).

Since KIM-1 is shed in tubular injury, we tried to subtract influences of AKI and CKD as well as (urogenital and) kidney infection through comparing levels of KIM-1 within the sepsis cohort.

Analysing only patients with known CKD, sKIM-1 levels between patients with sepsis and other critical illnesses did not differ (301.0 vs. 326.4 pg/mL, *p* = 0.773, Figure 2D). Of note, the presence of CKD did not alter the significance of KIM-1 elevation in sepsis, as patients with sepsis and neither known nor documented CKD (225.3 vs. 112.3 pg/mL, *p* = 0.009, Figure 2E) still presented with raised serum KIM-1 on admission day. Similarly, we investigated whether the presence of AKI influenced KIM-1 and found that patients with sepsis but no renal dysfunction (CKD and/or AKI after 48 h) had higher serum KIM-1 levels compared with non-sepsis patients without renal dysfunction (218.6 vs. 120.4 pg/mL, *p* < 0.019, Figure 2F) (defined as either serum creatinine increase ≥×1.5 or ≥0.3 mg/dL in the first 48 h, as established by the Kidney Disease: Improving Global Outcomes guidelines for acute kidney injury, KDIGO [41]).

Interestingly, urogenital sepsis showed the highest of all KIM-1 elevations (403.8 pg/mL, Table 2), but the increase remained statistically insignificant when compared with other infectious foci (403.8 vs. 218.8 pg/mL, *p* = 0.173, Figure 2G). To account for any bias caused by direct infectious injury to tubular cells, we compared levels of KIM-1 in patients with sepsis other than urosepsis (referred to as non-urosepsis) and patients without sepsis. We found a significant increase in KIM-1 levels (218.8 vs. 132.2 pg/mL, *p* = 0.031, Figure 2H).

### 2.5. Elevated sKIM-1 in Critically Ill Patients Mirrors Acute Kidney Dysfunction and Signals the Need for Renal Replacement Therapy

**Figure 3:**
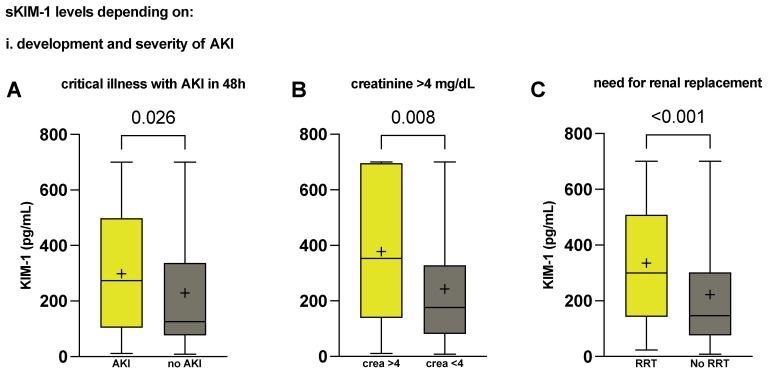
Characterisation of serum KIM-1 in patients with AKI. Serum KIM-1 concentrations on admission day in critically ill patients (**A**) that developed AKI KDIGO stage 1 within 48 h (n = 83 vs. 75); (**B**) that developed AKI KDIGO stage 3 (n = 28 vs. 164); or (**C**) needed RRT throughout ICU treatment (n = 50 vs. 130). *p* values reported are from the Mann–Whitney U test and were considered statistically significant at *p* < 0.05. Medians with first and third quartile are shown. Means are shown as +. Whiskers represent lowest and highest value. AKI, acute kidney injury. Crea, serum creatinine. KIM-1, kidney injury molecule-1. RRT, renal replacement therapy.

To investigate whether serum KIM-1 might allow assessment of acute kidney dysfunction, similar to urinary KIM-1 and alongside its elevation in both sepsis and critically ill patients who died in the ICU, we analysed critically ill patients who met the criteria for acute or acute-on-chronic renal injury (defined as a serum creatinine increase of at least 1.5 times or at least 0.3 mg/dL in the first 48 h, as established by the Kidney Disease: Improving Global Outcomes guidelines for acute kidney injury, KDIGO [41]). We found that critically ill patients with mild acute kidney injury (KDIGO AKI stage 1) had elevated serum KIM-1 levels compared with those without kidney injury (273.3 vs. 125.8 pg/mL, *p* = 0.026, Figure 3A).

The severity of kidney injury increased the significance level, as patients with a creatinine level of ≥4 mg/dL, equalling KDIGO AKI stage 3 (352.8 vs. 176.4 pg/mL, *p* = 0.008, Figure 3B), and patients with the need for immediate or later renal replacement therapy (RRT) (299.7 vs. 146.3 pg/mL, *p* < 0.001, Figure 3C) demonstrated significantly higher KIM-1 serum levels than patients without acute(-on-chronic) kidney injury.

### 2.6. sKIM-1 Correlates with Parameters of Inflammation and Liver, Renal, and Haematopoietic Function, as Well as Extent of Organ Failure

**Figure 4:**
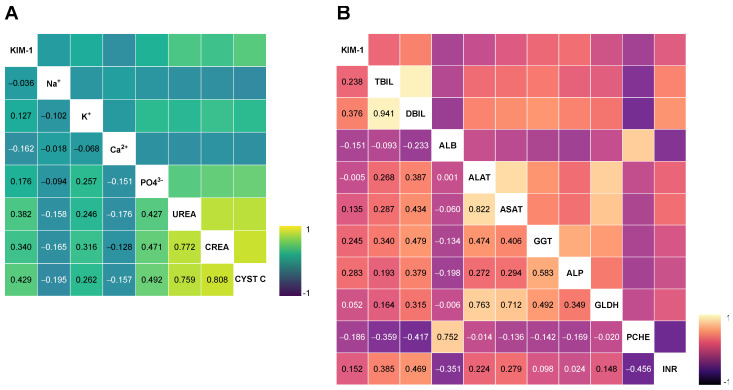
Spearman Rho coefficient heatmaps of correlations between KIM-1 and renal (**A**) and liver parameters (**B**) on admission day. ALB, albumin. ALAT, alanine aminotransferase. ALP, alkaline phosphatase. ASAT, aspartate aminotransferase. Ca^2+^, serum calcium. Crea, creatinine. Cyst C, cystatin C. DBIL, direct bilirubin (conjugated). GGT, gamma-glutamyl transferase. GLDH, glutamate dehydrogenase. INR, international normalised ratio. K^+^, Serum potassium. KIM-1, kidney injury molecule-1. Na^+^, serum sodium. PCHE, pseudocholinesterase. PO_4_^3−^, serum phosphate. TBIL, total bilirubin.

As the KIM-1 protein has been linked to sepsis and the development of acute renal dysfunction, our objective was to examine other potential factors that may influence KIM-1 serum concentrations. To do this, we conducted correlation analyses using a wide range of laboratory and clinical markers (Supplementary Table S1). A visual representation of the correlation with renal (A) and liver parameters (B) can be found in Figure 4.

We observed positive correlations with all established markers of inflammation (WBC Spearman’s r = 0.154 [*p* = 0.034], CRP r = 0.257 [*p* < 0.001], PCT r = 0.357 [*p* < 0.001]). Correspondingly, serum KIM-1 correlated inversely with parameters of haemodilution and haematopoietic function (haemoglobin Spearman’s r = −0.178 [*p* = 0.034] and platelets r = −0.181 [*p* = 0.012]). Unsurprisingly, serum KIM-1 also showed positive correlations with urea (Spearman’s r = 0.382 [*p* < 0.001]), creatinine (r = 0.340 [*p* < 0.001]), and cystatin C (r = 0.429 [*p* < 0.001]), as well as clinical parameters for renal dysfunction, such as the number of days spent on RRT r = 0.262 [*p* < 0.001]). In addition, serum KIM-1 demonstrated intercorrelations with markers for excretory liver function and cholestasis, such as bilirubin (r = 0.238 [*p* < 0.001]), GGT (r = 0.245 [*p* < 0.001]), ALP (r = 283 [*p* < 0.001]), and the INR (r = 0.152 [*p* = 0.039]). Interestingly, sKIM-1 also showed a correlation with the partial thromboplastin time (pTT r = 0.220 [*p* = 0.003]).

Lastly, serum KIM-1 on admission day showed significant correlations with scoring systems for disease and multi-organ dysfunction (SOFA r = 0.389 [*p* = <0.001], APACHE II r = 0.224 [*p* = 0.005]).

### 2.7. sKIM-1 Increase in Critically Ill Patients Is Multimodal

To further reflect on the multi-organ correlations, we investigated sKIM-1 stratified along severity of organ dysfunction, dissecting the individual components of the SOFA score.

**Figure 5:**
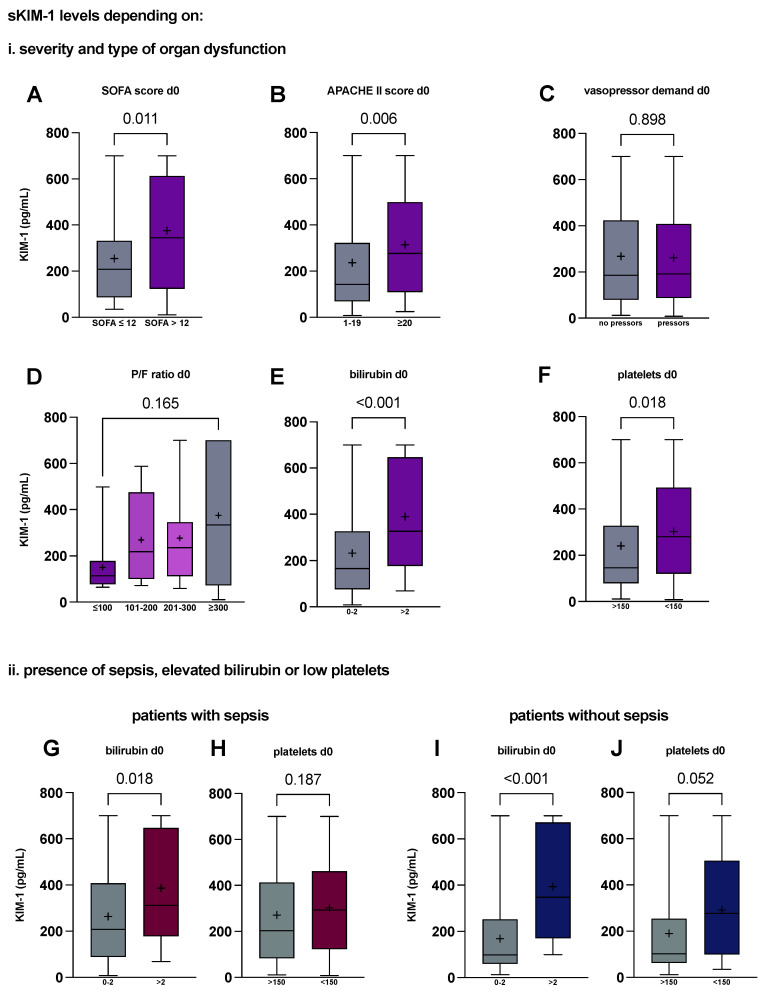
Serum KIM-1 is elevated in patients with multi-organ dysfunction, correlating with liver and haematopoietic function. Serum KIM-1 concentrations on admission day (day 0) in critically ill patients that (**A**) presented with multi-organ dysfunction and SOFA score > 12, compared with patients with SOFA ≤ 12 (n = 50 vs. 27) and (**B**) presented with multi-organ dysfunction and APACHE II score ≥ 20, compared with patients with APACHE II < 20 (n = 94 vs. 59). Singular components of the SOFA score had differential levels of KIM-1 elevation: (**C**) KIM-1 concentrations in patients in the ICU needing vasopressors therapy on day 0 compared with patients without (n = 115 vs. 70); (**D**) serum KIM-1 concentrations stratified along paO_2_/FiO_2_ (Horowitz index, P/F ratio); (**E**) dependent on total serum bilirubin levels (n = 155 vs. 37); and (**F**) grouped according to platelet concentration/nL (n = 68 vs. 124). The presence of sepsis had no influence on either bilirubin elevation or platelet count: Serum KIM-1 in sepsis, depending on (**G**) bilirubin levels (n = 104 vs. 21) and (**H**) platelet count (n = 78 vs. 45). Serum KIM-1 in patients without sepsis, again stratified for (**I**) bilirubin (n = 51 vs. 16) and (**J**) platelet count (n = 46 vs. 21). *p* values reported are from the Mann–Whitney U test and were considered statistically significant at *p* < 0.05. Medians with first and third quartile are shown. Means are shown as +. Whiskers represent lowest and highest value. APACHE II, acute physiology and chronic health evaluation II. SOFA, sequential organ failure assessment. P/F ratio, paO_2_/FiO_2_ ratio.

Patients with advanced sepsis and multiple organ dysfunction upon admission to the ICU, as determined by a SOFA score > 12 or an APACHE score ≥ 20, had significantly higher KIM-1 serum levels than their counterparts with lower scores (208.2 vs. 344 pg/mL, *p* = 0.011 and 143.3 vs. 277.3 pg/mL, *p* = 0.006, Figure 5A,B). Since renal dysfunction was a component in both scores for organ dysfunction, we sought to analyse all singular components and found no difference in serum KIM-1 concentrations in patients with vasopressor demand on admission day (Figure 5C). There was, however, an inverse trend towards increased serum KIM-1 depending on the P/F ratio (Horowitz on day 0, Figure 5D) and a significant difference between patients with bilirubin levels greater than 2 mg/dL (165.6 vs. 326.4 pg/mL, *p* < 0.001).

Furthermore, building on the negative correlation with platelet concentration, critically ill patients with platelet levels below 150/nL demonstrated significantly higher serum KIM-1 levels than their counterparts (279.4 vs. 146.6 pg/mL, *p* = 0.018).

To differentiate influences of septic biliary injury or thrombocytopenia, we further split groups into septic and non-septic patients and found no sepsis dependency for bilirubin (sepsis: 208.2 vs. 311.7 pg/mL, *p* = 0.018 Figure 5G; without sepsis: 98.2 vs. 347 pg/mL, *p* = <0.001, Figure 5I) or platelet count (sepsis: 203.3 vs. 293 pg/mL, *p* = 0.187, Figure 5H; without sepsis: 102.1 vs. 277.3 pg/mL, *p* = 0.052, Figure 5J). Of note, KIM-1 elevation came close to significance in patients without sepsis and thrombocytopenia <150/nL, but not sepsis.

### 2.8. Serum KIM-1 as Predictor for Disease Severity and Hospital-Related Outcomes

We utilised binary logistic regression models to assess the predictive ability of serum KIM-1 for various hospital-related outcomes. To test for independent associations of KIM-1, we performed different uni- and multivariate analyses, accounting for relevant factors that are known to be associated with sepsis and disease severity and that showed significant differences in prior analysis, i.e., CKD, COPD, bilirubin > 2 mg/dL, and platelets < 150/nL (Table 5). KIM-1 values demonstrated a wide range from 8.32 pg/mL to 700 pg/mL with multiple measurements at 700 pg/mL/. To better account for outliers and skewed distribution when evaluating associations of non-normally distributed biomarkers to binary outcomes, KIM-1 was analysed as log_e_-transformed values (LN, natural logarithm). Given the high intercorrelation with creatinine as a continuous variable and dichotomised to diagnose AKI, multivariate analyses with both variables were not performed.

The univariate odds ratio (OR) for the presence of sepsis at admission was 1.48 (95% confidence interval 1.08–2.02, *p* = 0.015, Table 5) per 1 log_e_ (pg/mL) increase in KIM-1, demonstrating a significant association. Interestingly, the association remained when adjusting for either comorbidities (CKD, COPD) or all described covariates (CKD, COPD, bilirubin, platelet count) at odds ratios of 1.47 (1.07–2.02, *p* = 0.017) and 1.60 (1.13–2.27, *p* = 0.008) per 1 log_e_ increase in sKIM-1, respectively.

Furthermore, sKIM-1 on the admission day became a relevant predictor in multivariate analysis for sepsis in patients without known CKD or AKI after 48 h when adjusting for COPD, bilirubin, and platelets (adjusted OR 1.89 [1.03–3.48] per log_e_ increase in KIM-1, *p* = 0.041, Table 5). Thus, the regression analysis mirrored the increase in sKIM-1 in these patients (Figure 2F).

Analysing AKI and accounting for outliers through logarithmisation led to a close but non-significant association in univariate analysis, with an OR of 1.48 per 1 log_e_ increase in KIM-1 (0.96–2.29, *p* = 0.078). Interestingly, when adjusting for only CKD and COPD, the association became significant (OR 1.60 [1.01–2.53], *p* = 0.046). Further accounting for bilirubin and platelets, the significance level was lost (OR 1.51 [0.94–2.44], *p* = 0.091).

Building on this, sKIM-1 remained individually associated with the need for RRT throughout univariate and all covariate analyses (adjusted odds ratio 2.08 [1.21–3.56] per 1 log_e_ increase, *p* = 0.008). KIM-1 on admission day was also independently able to predict multi-organ dysfunction (SOFA > 12, adjusted OR for CKD, COPD, bilirubin, and platelets 2.24 [1.05–4.78], *p* = 0.038 and APACHE ≥ 20, adjusted odds ratio 1.94 [1.27–2.96], *p* = 0.02 per 1 log_e_ KIM-1 increase, respectively).

Finally, even though sKIM-1 was close to being associated with death in the ICU when adjusting for CKD and COPD alone, it ultimately demonstrated no association with either short- (in the ICU) or long-term (360 days) mortality when adjusting for all mentioned covariates.

## 3. Discussion

While urinary KIM-1 has been characterised thoroughly in acute and chronic kidney injury [14,25,36], there are limited data on serum KIM-1 in these or other settings [35,37,42,43,44]. Given its implication as a non-myeloid scavenger receptor, facilitating semi-professional phagocytic reactions, investigating KIM-1 in sepsis and other critical illness may reduce the knowledge gap in SA-AKI and critical illness.

We demonstrated that circulating KIM-1 concentrations in this cohort were not influenced by demographic factors or important comorbidities, such as diabetes, hypertension, heart failure, or cardiovascular disease. However, KIM-1 serum concentrations were elevated in patients with sepsis and septic shock who were admitted to the ICU. Interestingly, even patients without CKD or AKI (renal dysfunction) and sepsis had higher levels of circulating KIM-1. This suggests that KIM-1 may have a role as a predictive biomarker. In fact, critically ill patients who died in the ICU without known CKD or AKI at day 2 showed significantly elevated peripheral KIM-1 concentrations.

Investigating all critically ill patients, blood KIM-1 correlated with both renal function (AKI) and the need for renal replacement therapy. Further demonstrating a link to multi-organ dysfunction, KIM-1 was significantly increased with higher SOFA and APACHE II scores, additionally rooted in the correlation with increased bilirubin and lower platelets.

Serum KIM-1 closely correlated with all established markers of renal dysfunction and showed links to parameters of cholestasis and haematopoietic function. Finally, circulating KIM-1 on the admission day was able to predict sepsis, multi-organ dysfunction, and the need for RRT, even when adjusting for relevant confounders. Additionally, sKIM-1 was elevated in patients with sepsis and neither reported CKD or AKI after 48 h. In these patients, KIM-1 was able to predict sepsis on day 0.

So far, only a few studies have investigated blood or urine levels of KIM-1 in relation to sepsis and critical illness.

A small, but interesting study investigated serum KIM-1 in a human endotoxin model through administering LPS and found significant elevation in both KIM-1 and creatinine in response to the inflammatory stimulus [39]. That study did not investigate liver injury.

Another study in paediatric patients reported that urine levels of KIM-1 can predict AKI in septic children [43].

Of note, that study in children included a wide variety of patient composition, with ages ranging from one month to 18 years. Another publication investigated urinary KIM-1 combined with netrin-1 in the early diagnosis of SA-AKI and found similar results [42].

A recent Chinese study investigated both urinary and serum levels of KIM-1 and neutrophil gelatinase-associated lipocalin (NGAL) in SA-AKI. That study found that serum levels of KIM-1 were elevated in patients with sepsis who developed AKI. Somewhat in contrast to the current study, however, serum levels of KIM-1 were not able to predict the severity or prognosis of AKI and sepsis [44].

Sabbisetti et al. reported that circulating (plasma) KIM-1 served as a biomarker specifically reflecting AKI and CKD in tubular injury and demonstrated elevated blood KIM-1 parameters in animal models of kidney injury (e.g., ischemia/reperfusion, nephrotoxicity) [35]. The authors noted that next to possible bloodstream release, a back-leak of tubular components into the circulation in tubular injury, influenced by changes to microvascular permeability, is likely [45,46]. To counter bias of KIM-1 homolog expression in the liver, the authors also comprehensively chose to perform single intraperitoneal injections of carbon tetrachloride (CCl_4_, a known hepatotoxic agent often used to induce hepatic fibrosis [47]) in mice to examine possible plasma KIM-1 changes attributed to (hepatocellular) liver injury. After 48 h, mice challenged with intraperitoneal CCl_4_ did not respond with plasma KIM-1 alterations. In that study, human plasma KIM-1 was not investigated in relation to liver injury.

Interestingly, a later, well-designed publication by the same authors investigated plasma KIM-1 in acetaminophen (APAP) overdose, APAP-induced liver injury, and APAP-induced acute liver failure (ALF) and found that plasma KIM-1 on admission day was superior to creatinine in predicting acute kidney injury in APAP-associated injury and failure [48]. In APAP overdose without liver injury, plasma KIM-1 was significantly lower than in patients with APAP-ALF that died or needed liver transplant. Peculiarly, even though survivors of APAP-induced liver injury demonstrated significantly higher transaminases than patients that had only APAP-overdosing and no transaminases, plasma KIM-1 reported by Antoine et al. did not differ significantly between the two groups [48].

Thus, the difference in the correlation with liver parameters observed in the current study may be attributed to prior studies having focused on hepato- rather than cholangiocellular damage (e.g., CCL_4_, APAP). Indeed, in this study, sKIM-1 concentrations did not correlate with transaminases, but rather with indicators of cholangiocellular injury, such as bilirubin, GGT, and AP.

Correlation with parameters of cholestasis might therefore very well be connected to patterns of KIM-1 expression. Portended by the arterial-only blood supply of the peribiliary vascular plexus, cholangiocellular injury caused by hypoperfusion and -oxaemia is far more common in sepsis than hepatocellular injury (possibly related to the dual blood supply) and is simultaneously the facilitator of secondary sclerosing cholangitis in critically ill patients (SSC-CIP/SSC) [49,50]. This correlation needs further investigation, as sKIM-1 remained elevated even in patients with bilirubinaemia without sepsis. Interestingly, due to the dependence of plasma coagulation on lipophile vitamins such phytonadione, serum KIM-1 correlation to the INR could also derive from cholestatic injury [51,52]. Remarkably, in cirrhosis, urinary KIM-1 levels increased with the Child–Pugh score and were associated with AKI secondary to cirrhosis decompensation [53].

Another consideration is the association of decreased excretory function (bilirubin) as a signal for advanced liver disease and portal hypertension. On a molecular level, portal hypertension in CLD is characterised by intestinal translocation of bacterial compounds such as LPS. Patients with cirrhosis, decompensation, and organ failure (so called acute-on-chronic liver failure, ACLF) portray disease-characterising immune dysregulation that, along with complications like hepatorenal syndrome, may present stimuli for higher KIM-1 release.

As KIM-1 has not been reported present on megakaryocytes or platelets, but rather in lymphatic hematopoietic lineages, the correlation to thrombocytopenia needs further investigation. We could not observe a link to portal hypertension-induced thrombopenia, but the association might very well be biased to reactivity, such as septic thrombopenia, drug association (especially anti-infective agents), and/or dialysis filters. Of note, in subgroup analysis, we were unable to find a correlation to septic thrombocytopenia. Another puzzling aspect is the inverse relation to the P/F ratio and COPD in this critically ill cohort, which indeed might be biased by the limited sub-cohort numbers.

This study has several limitations. Although conducting a single-centre study allows extensive cohort characterisation and low-threshold external replication, smaller recruitment numbers decrease statistical power. For example, KIM-1 correlated positively with NTproBNP, but the cohort included a limited number of patients with acute complications due to cardiovascular disease and cardiorenal syndrome, possible underestimating KIM-1 dynamics. Thus, in sub-cohort investigations, statistical relevance is decreased or impossible. The study population included a wide heterogeneity of disease and sepsis aetiologies, increasing the representative capacity, but generalisation remains questionable. Moreover, despite the extensive amount of clinical data collected and the long follow-up, our study is confined to portraying a limited time frame and a single-centre clinical approach.

A third limitation is the lack of serum KIM-1 measurements in a healthy control cohort. Even though KIM-1 in the proximal tubule is shed only in injury, we were unable to present comparisons between healthy individuals and critically ill patients.

Additionally, the chosen methods may have limited this extensive analysis. According to the study design, KIM-1 was measured on the day of admission, not repeatedly. Dynamics of KIM-1 levels might offer a better understanding through sequential assessments over the course of disease. Furthermore, the ELISA used for serum KIM-1 measurements applied a cut-off value of 700 pg/mL. Several measurements were situated at the cut-off level, raising the question of whether measurements above the threshold would provide a better understanding of serum KIM-1 distribution in critical illness. However, the overall distribution of measurements was comparable to those published relating to humans [35].

Possible confounders are also represented by the duality of KIM-1 homologs, as KIM-1 measurements in urine certainly represent KIM-1b. The second homolog, KIM-1a, is found on biliary cells and has been less well investigated but is likely to have played a major role in the findings of this study. Considering the pioneering findings in KIM-1 research, serum KIM-1 closely correlated with the development of AKI in the ICU. Serum KIM-1 concentrations on the admission day were therefore able to predict the need for renal replacement therapy.

Arguably, the most relevant finding of this current study is the association of sKIM-1 with the extent of multi-organ dysfunction, which in this cohort correlated with the multimodality of organ failure. Of note, this might fall in line with previous research that demonstrated a significant KIM-1 increase in a human endotoxin model [39]. KIM-1 was able to predict sepsis on the admission day, even when kidney dysfunction was not present at admission or after 48 h. Even though KIM-1 is tightly interwoven with renal perfusion, function, and injury, critically ill patients without known chronic kidney disease and/or AKI in the ICU presented with higher sKIM-1 levels on admission day. Non-survivors without kidney dysfunction after 48 h of ICU treatment had significantly higher KIM-1 serum concentrations on day 0. Although this increase was not transferable to an association with the long-term outcome of all critically ill patients, KIM-1 was able to predict sepsis even in patients with neither CKD nor AKI. This might signal a role in sepsis other than shedding from tubular cells. A connection may lie within the molecular ability to recognise PS on apoptotic leaflets, conferring signals for rapid clearance of apoptotic cells and possible shedding from hypoxemic biliary cells. The latter especially warrants further investigation. A different possibility is subclinical tubular kidney injury that does not transfer into retention of creatinine or that signals for incipient kidney dysfunction not reflected in current diagnostic criteria. If so, future research must further investigate the role of inapparent kidney injury in conveying multi-organ inflammatory response and organ crosstalk.

Notably, the intercorrelation of creatinine with KIM-1 in this and prior studies was high. Given the design of this study, the aim was not to conduct a superiority analysis for KIM-1. Adjusting for creatinine in multivariate models was therefore statistically inadequate for the goal of the study. We demonstrated that sKIM-1 concentrations in this cohort were independently associated with sepsis, severe kidney dysfunction, and disease severity. As adjusting for subcomponents of the SOFA and APACHE scores led KIM-1 to remain as a predictor for multi-organ dysfunction, correlation with AKI as a component of both scores might have remained. In this regard, diagnosis of chronic kidney dysfunction, also dependent on creatinine and itself associated with KIM-1, did not diminish predictive ability for disease severity.

A method to address potential confounders lies in repeated measurements of KIM-1 throughout the course of the disease, with normalisation of urine KIM-1 to serum KIM-1 to also account for associations with cholestatic markers and different homologs.

As these data suggest that KIM-1 might aid risk assessment in critical illness, future and prospective studies are needed to further validate and dissect these initial findings.

## 4. Materials and Methods

### 4.1. Study Design and Population

This observational cohort study examined critically ill adults (≥18 years) admitted to the medical intensive care unit (ICU) at University Hospital RWTH Aachen (n = 192), to investigate the role of KIM-1 in sepsis, septic shock, and other critically illness. Patients were included consecutively upon admission to the ICU and when the expected ICU stay exceeded 48 h, as previously described [54]. Patients under 18 years of age or with short-term ICU stays due to e.g., post-procedural observation periods, as well as patients transferred from another ICU, were excluded. Further exclusion criteria included pregnancy and prior psychiatric disorder that led to an inability to consent. Patient cohorts consisted of 125 patients with sepsis and 67 patients with critical illness other than sepsis. Sepsis was defined according to the Third International Consensus Definitions for Sepsis and Septic Shock (Sepsis-3) [1]. AKI was defined according to the Kidney Disease Improving Global Outcomes (KDIGO) criteria, with either serum creatinine increase ≥×1.5 or ≥0.3 mg/dL in the first 48 h [41]. Baseline creatinine was defined as the lowest serum creatinine level within the past year of medical records. In patients without prior medical records and without chronic kidney disease, baseline creatinine was defined as the patient’s serum creatinine on admission day. Dependent on the KDIGO definition, admission day was defined as day 0.

Patients, relatives, and/or the documented primary physician were contacted at intervals of 6 months for 2 years following transfer to standard care units to assess long-term survival.

All patients, next of kin, or appointed legal guardians gave written informed consent. The study protocol was approved by the local ethics committee (EK150/06) at RWTH Aachen University and conducted in accordance with the requirements set out by the 1964 Declaration of Helsinki.

### 4.2. KIM-1 and Routine Laboratory Measurements

Immediately upon admission to the ICU (day 0) and prior to any therapeutic measurements, blood samples were collected. Samples were then centrifuged at 4° Celsius for 10 min, and the resulting serum was collected and stored at −80 °C. Measurements of KIM-1 were conducted in accordance with the manufacturer’s instructions using a quantitative enzyme-linked immunosorbent assay (Human Serum TIM-1/KIM-1/HAVCR Immunoassay, catalogue number DSKM100, R&D Systems^®^, Inc., Minneapolis, MN, USA). KIM-1 measurements were not performed sequentially. KIM-1 measurements were performed by experienced personnel who were blinded to the clinical course and other laboratory parameters. All other parameters were determined through routine laboratory analysis.

### 4.3. Statistical Analysis

Data are expressed as medians with interquartile ranges or absolute numbers and frequencies unless stated otherwise. Data are visualised using box plots with medians and means for continuous variables. Differences between two groups were analysed via the Mann–Whitney U test or Chi-square test for unpaired samples, as the data were assumed to be outside the Gaussian distribution. Differences between more than two groups were examined using the Kruskal–Wallis *H* test or analysis of variance (ANOVA) with either Tukey’s or Dunn’s post -hoc comparison. The correlation of variables was examined using Spearman rank correlation (*r*). Binary logistic regression analysis was performed to calculate odds ratios (ORs) for hospitalisation-related outcomes and events, adjusting for relevant confounders. ORs are presented with the corresponding confidence intervals (95%). To counter skewed distribution, analyses were performed using log_e_-transformed values (natural logarithm to Euler’s number e) of continuous variables.

Statistical analysis was conducted using IBM SPSS version 29 (SPSS, Chicago, IL, USA). Data visualisation was performed using GraphPad Prism version 9 (GraphPad Software, San Diego, CA, USA). *p*-values reported are two-sided and were considered significant at <0.05. To ensure full transparency, we included all non-significant *p*-values in the figures and text.

## Figures and Tables

**Table 1:** Baseline characteristics in patients in the ICU with and without sepsis at admission (day 0).

		Admission Day (Day 0)		
	All (n = 192)	Sepsis (n = 125)	Non-Sepsis (n = 67)	*p* Value
Age [years]	65 (48; 74)	65 (49; 74)	63 (48; 74)	0.662
Female (n/%)	79 (41%)	47 (38%)	32 (48%)	0.226
Body weight [kg]	76.3 (66.1; 89.5)	77.6 (66.7; 90)	75 (65.5; 86.5)	0.441
BMI [kg/m^2^]	25.5 (23; 29.3)	25.6 (23.1; 29.3)	25.4 (22.5; 29.32)	0.742
*Concomitant conditions* COPD	25 (13%)	17 (14%)	8 (12%)	0.745
Heart failure	39 (20%)	24 (19%)	15 (22%)	0.602
Cardiovascular disease	62 (32%)	38 (30%)	24 (36%)	0.332
Diabetes mellitus	50 (26%)	33 (26%)	17 (25%)	0.773
Alcohol use disorder	24 (13%)	11 (8%)	13 (19%)	**0** **.018**
Chronic kidney disease	33 (17%)	24 (19%)	9 (13%)	0.303
Malignancies	23 (12%)	19 (15%)	4 (6%)	0.061
Chronic liver disease	20 (10%)	9 (7%)	11 (16%)	**0** **.047**
Hypertension	74 (39%)	46 (39%)	28 (42%)	0.381
CCI [points]	4 (3; 7)	4 (3; 7)	4 (2; 6)	0.296
Haemoglobin [mmol/L]		6.3 (5.7; 7.3)	6.7 (5.65; 6.7)	0.122
Haematocrit [%]		31 (28; 35)	33 (27; 40)	0.170
Platelets [/nL]		195.5 (98; 306)	207 (116; 301)	0.562
WBC [/nL]		13.8 (9.8; 22)	11.3 (8.6; 17.1)	**0** **.044**
LDH [µmol/L]		4.77 (3.33; 6.57)	4.27 (3.15; 7.58)	0.501
Bilirubin [µmol/L]		10.2 (6.8; 27.2)	13.6 (6.8; 32.7)	0.304
ALP [µmol/L]		1.48 (1.06; 2.19)	1.37 (1; 1.92)	0.305
GGT [µmol/L]		1 (0.4; 2.71)	0.91 (0.46; 2.38)	0.993
Amylase [µmol/L]		0.45 (0.27; 0.87)	0.53 (0.35; 0.91)	0.333
Lipase [µmol/L]		0 (0; 0.28)	0.18 (0; 0.9)	**0** **.024**
ALT [µmol/L]		0.42 (0.23; 1.03)	0.58 (0.27; 1.37)	0.089
AST [µmol/L]		0.63 (0.38; 1.43)	0.82 (0.43; 2.12)	0.087
Albumin [g/L]		25 (21; 30)	32.3 (27; 36.6)	**<0.** **001**
Glucose [mmol/L]		7.4 (5.3; 9.4)	8.2 (6.5; 12.6)	**0** **.003**
NTproBNP [pg/mL]		2123 (472.4; 10,223.0)	842.8 (233.9; 5340.5)	**0** **.027**
Creatinine [µmol/L]		132 (70.4; 272.8)	88 (70.4; 193.6)	0.142
Cystatin C [mg/L]		1.7 (1.14; 2.81)	1.39 (0.84; 2.49)	**0** **.006**
Sodium [mmol/L]		138.3 (143; 142)	139.0 (134.0; 142.0)	0.973
Potassium [mmol/L]		4.4 (4; 4.9)	4.4 (4.0; 5.1)	0.319
Calcium total [mmol/L]		1.95 (1.79; 2.08)	1.99 (1.87; 2.15)	**0** **.020**
Chloride [mmol/L]		106.0 (100.0; 110.0)	105.5 (98.75; 109.25)	0.199
Phosphate [mmol/L]		1.31 (0.94; 1.74)	1.27 (0.97; 1.66)	0.843
Urea [mg/dL]		71.5 (45.0; 112.0)	55.0 (39.0; 90.75)	**0** **.027**
Uric acid [mg/dL]		6.8 (4.3; 9.2)	6.85 (4.8; 9.1)	0.483
CRP [mg/L]		178 (104; 230)	17.3 (6; 92)	**<0.** **001**
PCT [ng/L]		2.2 (0.4; 24)	0.27 (0.1; 1.1)	**<0.** **001**
IL-6 [pg/mL]		255 (71; 1212)	91.5 (20; 290)	**0** **.001**
Prothrombin time (Quick)		71 (51; 84)	73 (45; 88)	0.450
INR		1.2 (1.1; 1.4)	1.2 (1.1; 1.5)	0.511
PTT [s]		33 (28; 41)	29 (26; 40)	0.082
Fibrinogen [g/L]		6.3 (4.2; 474)	4 (2.3; 350)	**0** **.010**
D-Dimer [µg/L]		2789 (1009; 5799)	1333 (623; 6250)	0.349
KIM-1 [pg/mL]		191.6 (85.8; 388.9)	132.2 (66.1; 302.3)	**0** **.019**

*p* values reported are from the Mann–Whitney U test or Fisher’s exact test and are two-sided. *p* values < 0.05 were considered significant and bold. Medians with first and third quartile or frequencies with percentages are shown. AHT, arterial hypertension. ALAT, alanine aminotransferase. ALP, alkaline phosphatase. ASAT, aspartate aminotransferase. CCI, Charlson comorbidity index. CLD, chronic liver disease. CKD, chronic kidney disease. COPD, chronic obstructive pulmonary disease. CRP, C-reactive protein. CVD, cardiovascular disease. DM, diabetes mellitus. GGT, gamma-glutamyl transferase. IL-6, interleukin 6. INR, international normalised ratio. LDH, lactate dehydrogenase. NTproBNP, N-terminal pro brain natriuretic peptide. PCT, procalcitonin. PTT, partial thromboplastin time. WBC, white blood count.

**Table 2:** Aetiology of critical illness, sepsis, and subgroup KIM-1 serum concentrations.

	Sepsis (n = 125)	Non-Sepsis (n = 67)	KIM-1 [pg/mL]	*p* Value
Pulmonary	69 (55.2%)		170.7 (86.2; 375.9)	0.204
Abdominal	19 (15.2%)		218.2 (80.5; 479.4)	
Urogenital	10 (8%)		403.8 (220.7; 512.9)	
Other	27 (21.6%)		297.4 (201.9; 441.9)	
Cardiovascular disease		13 (19.4%)	125.8 (42.5; 235.6)	**0.044**
Advanced liver disease		13 (19.4%)	281.5 (145.4; 641.9)	
Exacerbated COPD		10 (14.9%)	104.1 (83.2; 265.9)	
Other		31 (46.3%)	120.4 (54.2; 301.8)	

*p* values reported are from the Kruskal–Wallis test. *p* values < 0.05 were considered significant and bold. Medians with first and third quartile or frequencies with percentages are shown. KIM-1, kidney injury molecule-1.

**Table 3:** Intensive care scoring systems, clinical characteristics, and mortality in patients in the ICU with and without sepsis at admission (day 0) and on day 2.

	Admission Day (Day 0)		
	Sepsis (n = 125)	Non-Sepsis (n = 67)	*p* Value
SOFA score	11 (6.75; 14.25)	7 (3; 11)	**0.012**
SAPS II score	43 (34; 50)	41.5 (37.5; 54.75)	0.605
APACHE II score	18 (11; 24)	16 (8; 21)	**0.039**
SIRS (points)	2 (2; 2)	1 (0; 1)	**<0.001**
Mechanical ventilation (n)	92 (73.6%)	38 (56.7%)	**0.024**
Days of ventilation (n)	181 (0; 480)	27 (0; 176)	**0.002**
Noradrenalin (mg/kgKG/h)	59.35 (0; 179.3)	0 (0; 89.35)	**0.005**
RRT (n)	38 (30.4%)	12 (17.9%)	**0.045**
Days of RRT (n)	0 (0; 1)	0 (0; 0)	**0.042**
ICU days (n)	10 (5; 25)	6 (2; 13)	**<0.001**
Death in ICU (n)	38 (30.4%)	14 (20.9%)	0.214
30 days mortality (n)	41 (36.3%)	16 (30.8%)	0.606
1 year mortality (n)	65 (64.4%)	23 (50.0%)	0.142

*p* values reported are from the Mann–Whitney *U* test or Fisher’s exact test and are two-sided. *p* values < 0.05 were considered significant and bold. Medians with first and third quartile or frequencies with percentages are shown. APACHE II, acute physiology and chronic health evaluation II. ICU, intensive care unit. RRT, renal replacement therapy. SOFA, sequential organ failure assessment. SAPS II, simplified acute physiology score II.

**Table 4:** Comorbidities and their influence on serum KIM-1 concentrations at ICU admission.

Comorbidities	KIM-1 [pg/mL]	KIM-1 [pg/mL]c	*p* Value
w/o COPD (n = 25 vs. 167)	104.9 (85; 227.6)	218.7 (85.1; 424.4)	**0.035**
w/o heart failure yes (n = 39) vs. no (153)	279.2 (103.4; 407.9)	170.7 (80.7; 351.8)	0.109
w/o cardiovascular disease (n = 63 vs. 129)	176.5 (79.2; 337.7)	219.1 (89.4; 418.2)	0.256
w/o diabetes mellitus (n = 50 vs. 142)	208.4 (88.7; 338.4)	187.2 (80.1; 417.7)	0.812
w/o alcohol use disorder (n = 25 vs. 167)	176.3 (90.7; 509.4)	191.9 (83.3; 367.6)	0.618
w/o chronic kidney disease (n = 33 vs. 159)	301 (144.9; 508.6)	176.3 (80.9; 337.3)	**0.021**
w/o chronic liver disease (n = 20 vs. 172)	219.1 (106.6; 526.1)	187.2 (81.5; 364.7)	0.334
w/o hypertension (n = 75 vs. 117)	231.6 (91.1; 356)	176.3 (80; 418.2)	0.788

*p* values reported are from the Mann–Whitney U test. *p* values < 0.05 were considered significant and bold. Medians with first and third quartile or frequencies with percentages are shown. COPD, chronic obstructive pulmonary disease. KIM-1, kidney injury molecule-1.

**Table 5:** Associations between serum KIM-1 on admission day and parameters of clinical presentation and hospital-related outcomes.

Presence of sepsis on admission (day 0)	OR (95% CI)	*p* value
Unadjusted	1.48 (1.08–2.02)	0.015
Adjusted for CKD and COPD	1.47 (1.07–2.02)	0.017
Adjusted for CKD, COPD, bilirubin, and platelets	1.60 (1.13–2.27)	0.008
**Presence of sepsis on admission (day 0)** **in patients without CKD or AKI**	**OR (95% CI)**	***p* value**
Unadjusted	1.74 (0.997–3.04)	0.051
Adjusted for COPD	1.75 (1.01–3.06)	0.048
Adjusted for COPD, bilirubin, and platelets	1.89 (1.03–3.48)	0.041
**AKI after 48 h**	**OR (95% CI)**	***p* value**
Unadjusted	1.48 (0.96–2.29)	0.078
Adjusted for CKD and COPD	1.60 (1.01–2.53)	0.046
Adjusted for CKD, COPD, bilirubin, and platelets	1.51 (0.94–2.44)	0.091
**Need for RRT**	**OR (95%) CI)**	***p* value**
Unadjusted	2.20 (1.32–3.64)	0.002
Adjusted for CKD and COPD	2.11 (1.25–3.55)	0.005
Adjusted for CKD, COPD, bilirubin, and platelets	2.08 (1.21–3.56)	0.008
**MOD with SOFA > 12 points**	**OR (95%) CI)**	***p* value**
Unadjusted	2.53 (1.24–5.16)	0.011
Adjusted for CKD and COPD	2.44 (1.20–4.95)	0.014
Adjusted for CKD, COPD, bilirubin, and platelets	2.24 (1.05–4.78)	0.038
**MOD with APACHE** **≥ 20 points**	**OR (95%) CI)**	***p* value**
Unadjusted	1.76 (1.20–2.57)	0.003
Adjusted for CKD and COPD	1.84 (1.23–2.74)	0.003
Adjusted for CKD, COPD, bilirubin, and platelets	1.94 (1.27–2.96)	0.002
**Death in the ICU**	**OR (95%) CI)**	***p* value**
Unadjusted	1.28 (0.91–1.81)	0.154
Adjusted for CKD and COPD	1.34 (0.97–2.01)	0.070
Adjusted for CKD, COPD, bilirubin, and platelets	1.29 (0.87–1.91)	0.209
**Death at 360 days**	**OR (95%) CI)**	***p* value**
Unadjusted	1.29 (0.90–1.82)	0.165
Adjusted for CKD and COPD	1.34 (0.97–2.0)	0.076
Adjusted for CKD, bilirubin, and platelets	1.25 (0.85–1.84)	0.262

Odds ratios are based on binary logistic regression analyses using log_e_-transformed values. Covariates for adjusted odds ratios are taken from the day of admission (laboratory data from the same timepoint), data from prior hospitalisations, or follow-up survival data of the individual patients. Bilirubin signals for adjusting to a dichotomised categorical variable of below or higher than 2 mg/dL, platelets for dichotomisation into below or higher than 150/nL. Odds ratios are reported with the 95% confidence interval. *p* values were considered statistically significant at *p* < 0.05 AKI, acute kidney injury. APACHE II, acute physiology and chronic health evaluation II. CKD, chronic kidney disease. ICU, intensive care unit. ICU, intensive care unit. KIM-1, kidney injury molecule-1. MOD, multi-organ dysfunction. OR, odds ratio. RRT, renal replacement therapy. SOFA, sequential organ failure assessment.

## Data Availability

The datasets used and/or analysed during the current study are available from the corresponding author on reasonable request.

## Supplementary Materials

The following supporting information can be downloaded at: https://www.mdpi.com/article/10.3390/ijms25115819/s1.
