# Supplementary material for: Elevated Serum KIM-1 in Sepsis Correlates with Kidney Dysfunction and the Severity of Multi-Organ Critical Illness
Source: Int J Mol Sci. 2024 May 27;25(11):5819. doi: 10.3390/ijms25115819 (PMC11172102; doi:10.3390/ijms25115819)
Supplement: Supplementary file 1 [file ijms-25-05819-s001.zip › ijms-2998377-supplementary.pdf]

sKIM-1 levels depending on:

i. known comorbidities

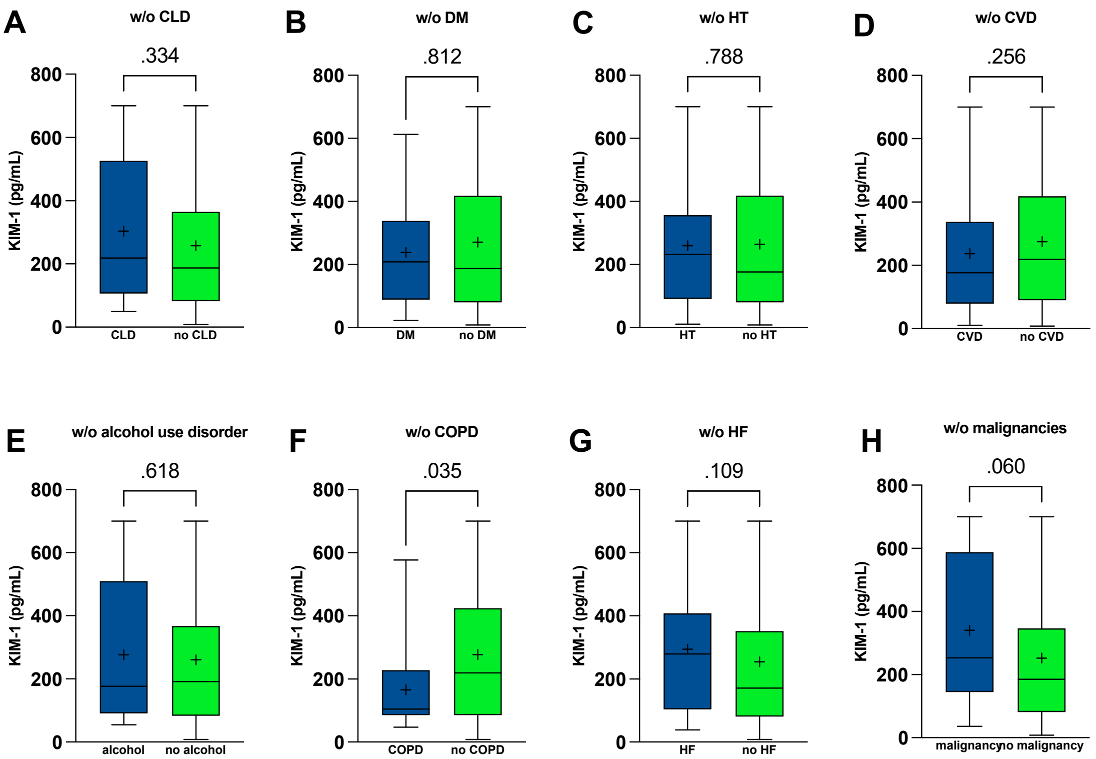

Supplemental Figure S1. Serum KIM-1 levels depending on known comorbidities at the time of admission: In patients with **(A)** chronic liver disease, **(B)** diabetes mellitus, **(C)** hypertension, **(D)** cardiovascular disease, **(E)** alcohol use disorder, **(F)** chronic obstructive pulmonary disease, **(G)** heart failure, **(H)** malignancies compared to those without. *p* values reported are from Mann-Whitney U test and were considered statistically significant at *p*<.05. Medians with first and third quartile are shown. Means are shown as +. Whiskers represent lowest and highest value. *CVD, Cardiovascular disease. CLD, Chronic liver disease. COPD, Chronic obstructive pulmonary disease. DM, diabetes mellitus. HF, heart failure. HT, hypertension. W/o, with/without.*

Supplemental Table S1. Correlations of different clinical and laboratory parameters with KIM-1 serum concentrations at ICU admission.

Parameters	r	*P* value
Demographics		
Age	.069	.312
Body mass index	.211	.115
Blood count and markers of inflammation		
White blood cells	.154	**.034**
Haemoglobin	-.178	**.014**
Platelets	-.181	**.012**
C-reactive protein	.257	**<.001**
Procalcitonin	.357	**<.001**
Interleukin 6	-.075	.361
Electrolytes and renal system		
Sodium	-.036	.619
Potassium	.127	.078
Calcium, total	-.162	**.025**
Phosphate	.176	**.019**
Urea	.382	**<.001**
Creatinine	.340	**<.001**
Cystatin C	.429	**<.001**
Ureic acid	.169	**.035**
Days of RRT	.262	**<.001**
Cardiopulmonary system and fluid balance		
NTproBNP	.314	**.002**
Vasopressor demand (day 0)	.117	.552
Diuresis (day 0)	-.059	.420
Net fluid balance (day 0)	.109	.139
Hepato-biliary system and coagulation		
Albumin	-.151	.089
Bilirubin, total	.238	**<.001**
Alanine amino transferase (ALAT)	-.005	.944
Aspartate amino transferase (ASAT)	.135	.072
Gamma-glutamyl transferase (GGT)	.245	**<.001**
Alkaline phosphatase (ALP)	.283	**<.001**
Lipase	.024	0.944
INR	.152	**.039**
pTT	.220	**.003**
Prothrombin time (Quick)	-.163	**.026**
Scoring systems and ICU		
SOFA score (day 0)	.389	**<.001**
SAPS II (day 0)	.165	.123
APACHE II (day 0)	.224	**.005**
Days on the ICU	.025	.728

APACHE II, Acute physiology and chronic health evaluation II. ICU, Intensive care unit. INR, International normalized ratio. NTproBNP, N-terminal prohormone of brain natriuretic peptide. pTT, Partial thromboplastin time. RRT, Renal replacement therapy. SOFA, Sequential organ failure assessment. SAPS II, Simplified acute physiology score II.
